# Risk stratification of stage II rectal mucinous adenocarcinoma to predict the benefit of adjuvant chemotherapy following neoadjuvant chemoradiation and surgery

**DOI:** 10.3389/fonc.2024.1352660

**Published:** 2024-03-05

**Authors:** Yahang Liang, Hualin Liao, Haoran Shi, Tao Li, Yaxiong Liu, Yuli Yuan, Mingming Li, Aidi Li, Yang Liu, Yao Yao, Taiyuan Li

**Affiliations:** ^1^ Department of General Surgery, The First Affiliated Hospital, Jiangxi Medical College, Nanchang University, Nanchang, Jiangxi, China; ^2^ Gastrointestinal Surgical Institute, Nanchang University, Nanchang Jiangxi, China; ^3^ Jiangxi Medical College, Nanchang University, Nanchang, Jiangxi, China

**Keywords:** neoadjuvant chemoradiation, stage II rectal mucinous adenocarcinoma, adjuvant chemotherapy, nomogram, survival

## Abstract

**Background:**

The treatment strategy for stage II rectal mucinous adenocarcinoma (RMA) recommends neoadjuvant chemoradiotherapy (NCR) followed by total mesorectal excision (TME). However, the necessity of adjuvant chemotherapy (AC) remains controversial.

**Materials and methods:**

Chi-square test was used to assess the relationship between pathological classification, AC and clinicopathological characteristics. Kaplan-Meier (KM) curves and the log-rank test were utilized to analyze differences in overall survival (OS) and cancer-specific survival (CSS) among different groups. Cox regression identified prognostic factors. Nomogram was established utilizing the independent prognostic factors. X-tile divided patients into three risk subgroups.

**Results:**

Compared to RMA, rectal adenocarcinoma (RA) demonstrates longer OS and CSS in all and non-AC stage II patients, with no difference in OS and CSS for AC stage II patients. Propensity score matching analyses yielded similar results. Stratified analysis found that AC both improve OS of RA and RMA patients. Age, gender, pathologic T stage, regional nodes examined, and tumor size were identified as independent prognostic factors for RMA patients without AC. A nomogram was constructed to generate risk scores and categorize RMA patients into three subgroups based on these scores. KM curves revealed AC benefits for moderate and high-risk groups but not for the low-risk group. The external validation cohort yielded similar results.

**Conclusions:**

In summary, our study suggests that, compared to stage II RA patients, stage II RMA patients benefit more from AC after NCR. AC is recommended for moderate and high-risk stage II RMA patients after NCR, whereas low-risk patients do not require AC.

## Introduction

Colorectal cancer is an extremely aggressive and fatal disease (1), with rectal cancer accounting for 48.3% of cases (2). Rectal mucinous adenocarcinoma (RMA) is a distinct pathological subtype of rectal cancer, characterized by the presence of extracellular mucinous components comprising more than 50% of the tumor volume (3). Numerous studies have indicated that RMA is associated with poor prognosis (4, 5), which is closely associated with a higher proportion of lymph node infiltration, peritoneal implantation, and larger tumor volumes (6, 7). Nonetheless, the treatment strategies for RMA and rectal cancer are similar, advocating for neoadjuvant chemoradiation (NCR) before total mesorectal excision (TME) surgery, followed by standard adjuvant chemotherapy (AC) (8). However, there is still ongoing debate about whether AC treatment is necessary for stage II RMA after surgery (9).

Studies indicate that the high presence of acidic mucus in RMA can unevenly distribute drugs, reducing their effectiveness, making RMA less responsive to chemotherapy (10). However, clinical research from various centers debates whether AC benefits RMA patients. Das et al.’s study with 562 rectal cancer cases undergoing AC found no relationship between the histological subtype of mucinous adenocarcinoma and AC sensitivity (11). Zhang et al. conducted a meta-analysis on the prognosis of locally advanced rectal cancer patients after receiving first-line AC. They found that RMA patients are insensitive to AC and have a poorer prognosis (12). Our population-based retrospective cohort study suggests that, compared to stage II RA, stage II RMA benefits more from AC after NCR. However, the application of AC is likely to introduce toxicity implications for patients. So, selectively administering AC to patients at high risk of distant recurrence could maximize the avoidance of unnecessary toxicity for patients.

Therefore, based on the clinicopathological characteristics of stage II RMA patients obtained from the Surveillance, Epidemiology, and End Results (SEER) database, we constructed a nomogram. Using the risk scores obtained from this nomogram, we categorized patients into three subgroups: high, moderate, and low risk. This risk stratification system provides guidance on whether AC is necessary for stage II RMA patients.

## Patients and methods

### Data source and patient selection

All patient data in this study were extracted from the SEER database (https://seer.cancer.gov/). We conducted the subsequent analysis using the most recent database, which is the incidence - SEER Research Plus Data, 17 Registries, Nov 2021 Sub (2000-2019). A total of 5549 patients were enrolled to our study. The criteria for inclusion were as follows: (1) primary rectal cancer, (2) the pathological diagnosis was confirmed as rectal adenocarcinoma (RA) or RMA (ICD-O-3: 8140/2, 8140/3, 8480/3, 8481/3), (3) received curative resection, (4) patients with stage II pathology, (5) patients who received radiotherapy and systemic chemotherapy before surgery. The criteria for exclusion were as follows: (1) did not receive NCR, (2) clinicopathologic information or follow-up data is incomplete. Ultimately, we collected the following information from the enrolled patients: age, gender, race, marital status, household income, pathological T stage, RNE, and tumor size. In addition, a validation cohort comprising 143 RMA patients recruited from the First Affiliated Hospital of Nanchang University from January 2010 to October 2018 was selected. This study was approved by the Ethics Committee of First Affiliated Hospital of Nanchang University. Each participant was signed informed consent forms.

### Subgroup and definition

Firstly, patients were divided into RA or RMA groups by the pathological classification. Then, based on whether they had received AC, each group was further divided into two groups, the AC group and the non-AC group. In addition, all RMA patients were divided into low (risk score: < 135, n = 132), moderate (risk score: 136-182, n = 107), and high-risk (risk score: > 182, n = 104) subgroups to evaluate the benefit of AC among different risk subgroups.

### Statistical analysis

The chi-square test was used to assess the relationship between pathological classification, AC and clinicopathological characteristics. Kaplan-Meier (KM) curves and the log-rank test were utilized to analyze differences in OS and cancer-specific survival (CSS) among different groups. Univariate and multivariate Cox regression analysis were employed to identify independent prognostic factors. Nomogram was constructed utilizing the independent prognostic factors identified through multivariate Cox regression analysis. The effectiveness of the nomogram was assessed for its discriminative ability, which was quantified by the concordance index (C-index). A calibration curve was produced to demonstrate the concordance between predicted and actual survival rate. Using X-tile software, all RMA patients were divided into three risk subgroups based on their nomogram risk scores. The statistical analyses in this study were conducted using SPSS software (version 25, IBM, Armonk, NY, USA) and R software (version 4.1.1). *P* < 0.05 was considered to indicate a statistically significant difference.

## Results

### Clinicopathological characteristics

**Figure 1:**
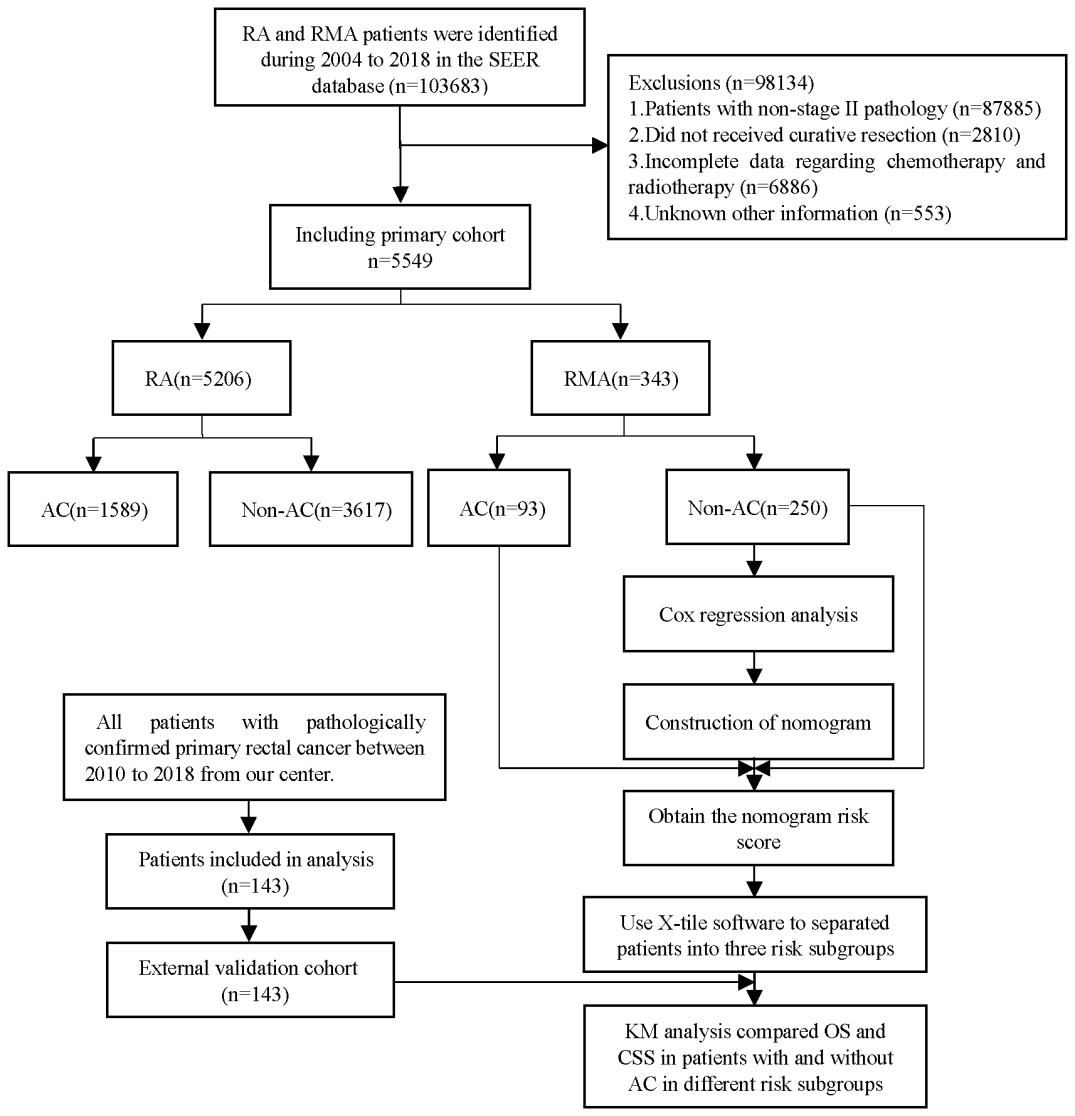
The workflow of the study cohort.

Between 2000 and 2019, according to our screening criteria, a total of 5549 patients were enrolled, with 5206 being RA and 343 being RMA. In the RA group, a total of 1589 patients received AC, while 3,617 did not receive AC. In the RMA group, 93 patients received AC, while 250 did not receive AC. **Figure 1** displays the flowchart depicting the selection process. Patients with RMA tend to have an advanced T stage (*P* < 0.001), fewer RNE (*P* < 0.001), and larger tumor size (*P* = 0.001) compared to patients with RA (**Table 1**). There are no differences in terms of gender, race, marital status, household income, pathological T stage and RNE (**Table 1**).

**Table 1:** Baseline characteristics of pathologic stage II patients with RA and RMA.

Variables	RA[n(%)]n=5206	RMA[n(%)]n=343	*P* value
Age			0.412
<65	3137(60.3%)	199(58.0%)	
≥65	2069(39.7%)	144(42.0%)	
Gender			0.933
Female	1985(38.1%)	130(37.9%)	
Male	3221(61.9%)	213(62.1%)	
Race			0.994
Non-white	957(18.4%)	63(18.4%)	
White	4249(81.6%)	280(81.6%)	
Marital status			0.837
Single	845(16.2%)	53(15.5%)	
Married	4187(80.4%)	280(81.6%)	
Unknown	174(3.3%)	10(2.9%)	
Household income			0.141
emsp <$65,000	2549(49.0%)	182(53.1%)	
emsp ≥$65,000	2657(51.0%)	161(46.9%)	
Pathological T			**< 0.001**
T3	4565(87.7%)	266(77.6%)	
T4	641(12.3%)	77(22.4%)	
Adjuvant chemotherapy			0.183
Non-AT	3617(69.5%)	250(72.9%)	
AT	1589(30.5%)	93(27.1%)	
RNE			**< 0.001**
<12	1978(38.0%)	166(48.4%)	
≥12	3228(62.0%)	177(51.6%)	
Tumor size			**0.001**
<5	2134(41.0%)	159(46.3%)	
≥5	1302(25.0%)	112(32.7%)	
Unknown	1770(34.0%)	72(21.0%)	

*P* values in bold indicate *P* < 0.05.

### Survival prognostic factor

**Figure 2:**
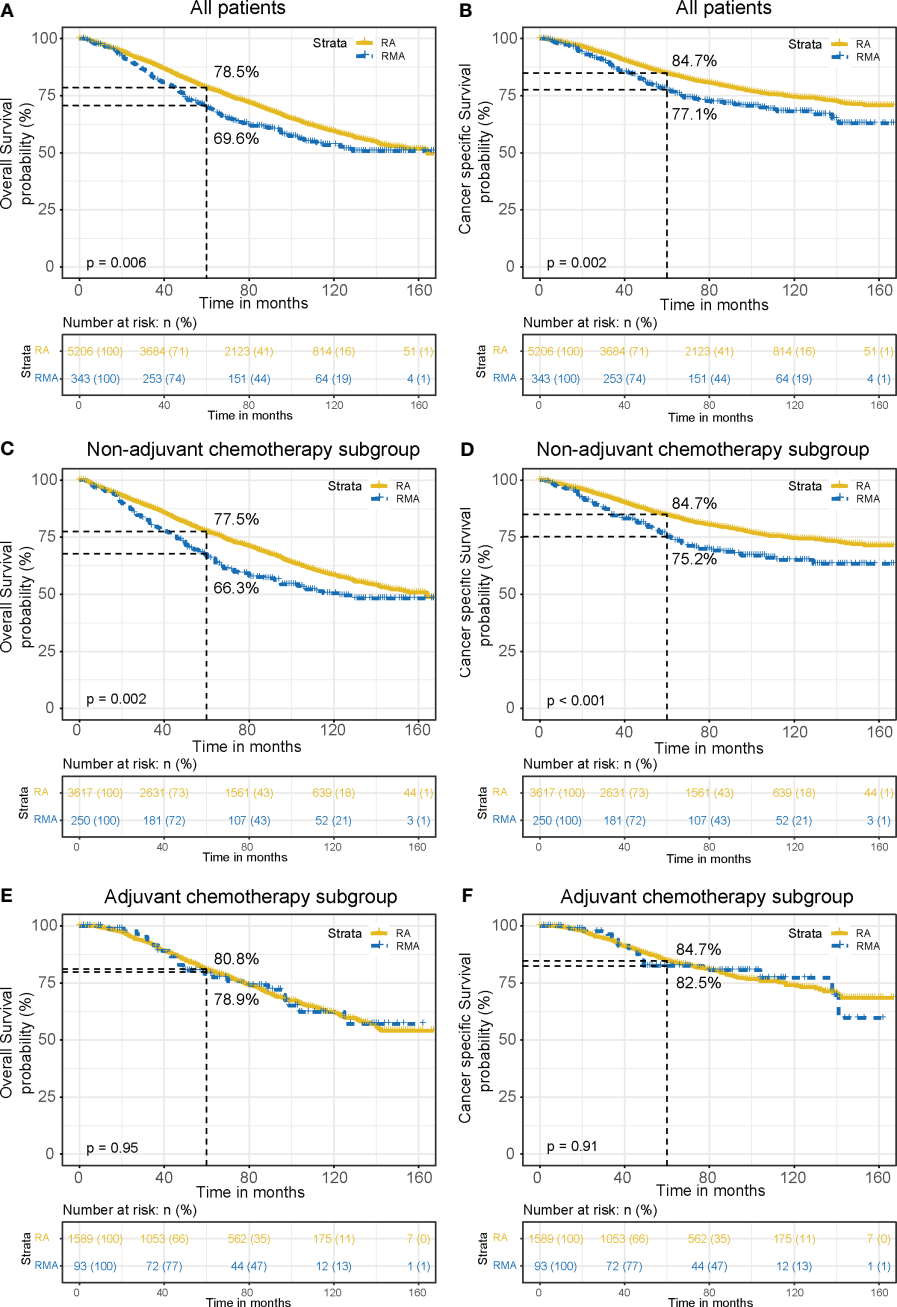
OS and CSS curves between the RA group and RMA group. OS **(A)** and CSS **(B)** for all patients. OS **(C)** and CSS **(D)** for non-AC patients. OS **(E)** and CSS **(F)** for AC patients.

Then, we performed the survival analysis and found that OS (5-year OS 78.5% vs. 69.6%, *P* = 0.006, **Figure 2A**) and CSS (5-year CSS 84.7% vs. 77.1%, *P* = 0.002, **Figure 2B**) were superior in RA group compared to the RMA group. Further subgroup analysis showed that in the patients without AC, the OS (5-year OS 77.5% vs. 66.3%, *P* = 0.002, **Figure 2C**) and CSS (5-year CSS 84.7% vs. 75.2%, *P* < 0.001, **Figure 2D**) of the RA group were better than those of the RMA group, but in the patients that received AC, there was no difference in OS (5-year OS 80.8% vs. 78.9%, *P* = 0.95, **Figure 2E**) and CSS (5-year CSS 84.7% vs. 82.5%, *P* = 0.91, **Figure 2F**) between the RA group and the RMA group. The above results indicate that RMA patients have a poorer prognosis compared to RA patients. However, AC could bridge the gap in prognosis between the two groups.

Cox regression analysis showed that age (*P* < 0.001), gender (*P* < 0.001), household income (*P* < 0.001), RNE (*P* < 0.001) and pathological classification (*P* = 0.017) were independent prognostic factors of OS (**Table 2**). Age (*P* < 0.001), marital status (*P* = 0.002), household income (*P* = 0.004), RNE (*P* < 0.001), pathological classification (*P* = 0.004) were independent prognostic factors of CSS (**Supplementary Table 1**).

**Table 2:** Univariate and multivariate Cox regression analyses of OS for the stage II patients with RA and RMA.

Variables	Univariate analysis		Multivariate analysis	
	HR (95% CI)	*P* value	HR (95% CI)	*P* value
Age		**< 0.001**		**< 0.001**
<65	Reference		Reference	
≥65	2.045 (1.854-2.256)		2.010 (1.821-2.218)	
Gender		**0.001**		**< 0.001**
Female	Reference		Reference	
Male	1.185 (1.069-1.313)		1.197 (1.080-1.326)	
Race		0.358		
Non-white	Reference			
White	1.062 (0.934-1.209)			
Marital status				
Single	Reference			
Married	0.905 (0.792-1.033)	0.139		
Unknown	0.983 (0.730-1.324)	0.909		
Household income		**< 0.001**		**< 0.001**
emsp <$65,000	Reference		Reference	
emsp ≥$65,000	0.814 (0.738-0.897)		0.803 (0.728-0.885)	
Pathologic T		0.188		
T3	Reference			
T4	1.100 (0.955-1.268)			
Adjuvant chemotherapy		**0.002**		0.204
Non-AT	Reference		Reference	
AT	0.835 (0.745-0.935)		0.928 (0.828-1.041)	
RNE		**< 0.001**		**< 0.001**
<12	Reference		Reference	
≥12	0.729 (0.662-0.804)		0.757 (0.687-0.835)	
Pathological classification		**0.006**		**0.017**
RA	Reference		Reference	
RMA	1.283 (1.075-1.531)		1.240 (1.039-1.480)	
Tumor size				
<5	Reference			
≥5	0.996 (0.889-1.116)	0.943		
Unknown	0.943 (0.831-1.070)	0.360		

*P* values in bold indicate *P* < 0.05.

### Propensity score matching

**Figure 3:**
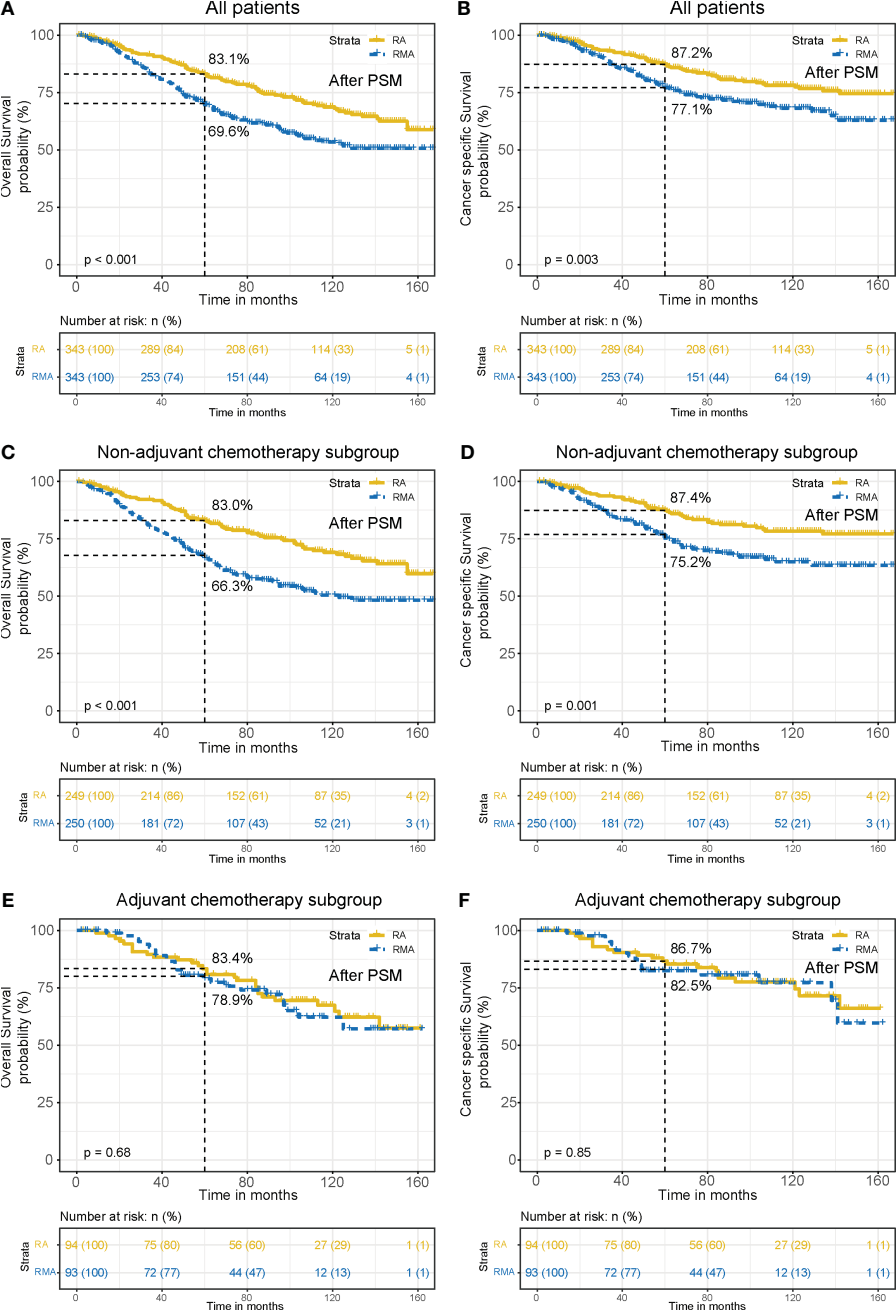
OS and CSS curves between the RA group and RMA group after PSM. OS **(A)** and CSS **(B)** for all patients. OS **(C)** and CSS **(D)** for non-AC patients. OS **(E)** and CSS **(F)** for AC patients.

In order to mitigate bias caused by confounding variables, we conducted a repeated analysis using propensity score matching (PSM). After adjustment with PSM, all variables were well balanced and no significant differences were found between the two groups (**Supplementary Table 2**). KM survival analysis showed that OS (5-year OS 83.1% vs. 69.6%, *P* < 0.001, **Figure 3A**) and CSS (5-year CSS 87.2% vs. 77.1%, *P* = 0.003, **Figure 3B**) were superior in RA group compared to the RMA group, consistent with the results before PSM. Subgroup analysis results also align with the pre-PSM findings. In the patients without AC, RA patients have better OS (5-year OS 83.0% vs. 66.3%, *P* < 0.001, **Figure 3C**) and CSS (5-year CSS 87.4% vs. 75.2%, *P* = 0.001, **Figure 3D**) than RMA patients. However, in the patients that received AC, there are no significant differences in OS (5-year OS 83.4% vs. 78.9%, *P* = 0.68, **Figure 3E**) and CSS (5-year CSS 86.7% vs. 82.5%, *P* = 0.85, **Figure 3F**) between two groups.

After PSM, Cox regression analysis showed that age (*P* < 0.001), RNE (*P* < 0.001) and pathological classification (*P* < 0.001) were independent prognostic factors of OS (**Table 3**). RNE (*P* < 0.001), pathological classification (*P* = 0.003) were independent prognostic factors of CSS (**Supplementary Table 3**). This further corroborates the reliability of the conclusion that RMA patients have a worse prognosis than RA patients, but AC could bridge the gap in the prognoses of the two groups.

**Table 3:** Univariate and multivariate Cox regression analyses of OS for the stage II patients with RA and RMA after PSM.

Variables	Univariate analysis		Multivariate analysis	
	HR (95% CI)	*P* value	HR (95% CI)	*P* value
Age		**< 0.001**		**< 0.001**
<65	Reference		Reference	
≥65	1.797 (1.387-2.328)		1.754 (1.351-2.278)	
Gender		0.357		
Female	Reference			
Male	1.135 (0.867-1.487)			
Race		0.144		
Non-white	Reference			
White	1.312 (0.917-1.887)			
Marital status				
Single	Reference			
Married	0.860 (0.600-1.233)	0.411		
Unknown	0.377 (0.116-1.227)	0.105		
Household income		0.123		
<$65,000	Reference			
≥$65,000	0.816 (0.630-1.057)			
Pathologic T		0.220		
T3	Reference			
T4	1.218 (0.889-1.670)			
Adjuvant chemotherapy		0.216		
Non-AT	Reference			
AT	0.825 (0.609-1.119)			
RNE		**< 0.001**		**< 0.001**
<12	Reference		Reference	
≥12	0.544 (0.417-0.709)		0.580 (0.444-0.757)	
Pathological classification		**< 0.001**		**< 0.001**
RA	Reference		Reference	
RMA	1.657 (1.276-2.150)		1.710 (1.317-2.221)	
Tumor size				
<5	Reference			
≥5	0.913 (0.677-1.231)	0.549		
Unknown	1.007 (0.717-1.415)	0.968		

*P* values in bold indicate *P* < 0.05.

### Pathological classification-stratified analysis

**Figure 4:**
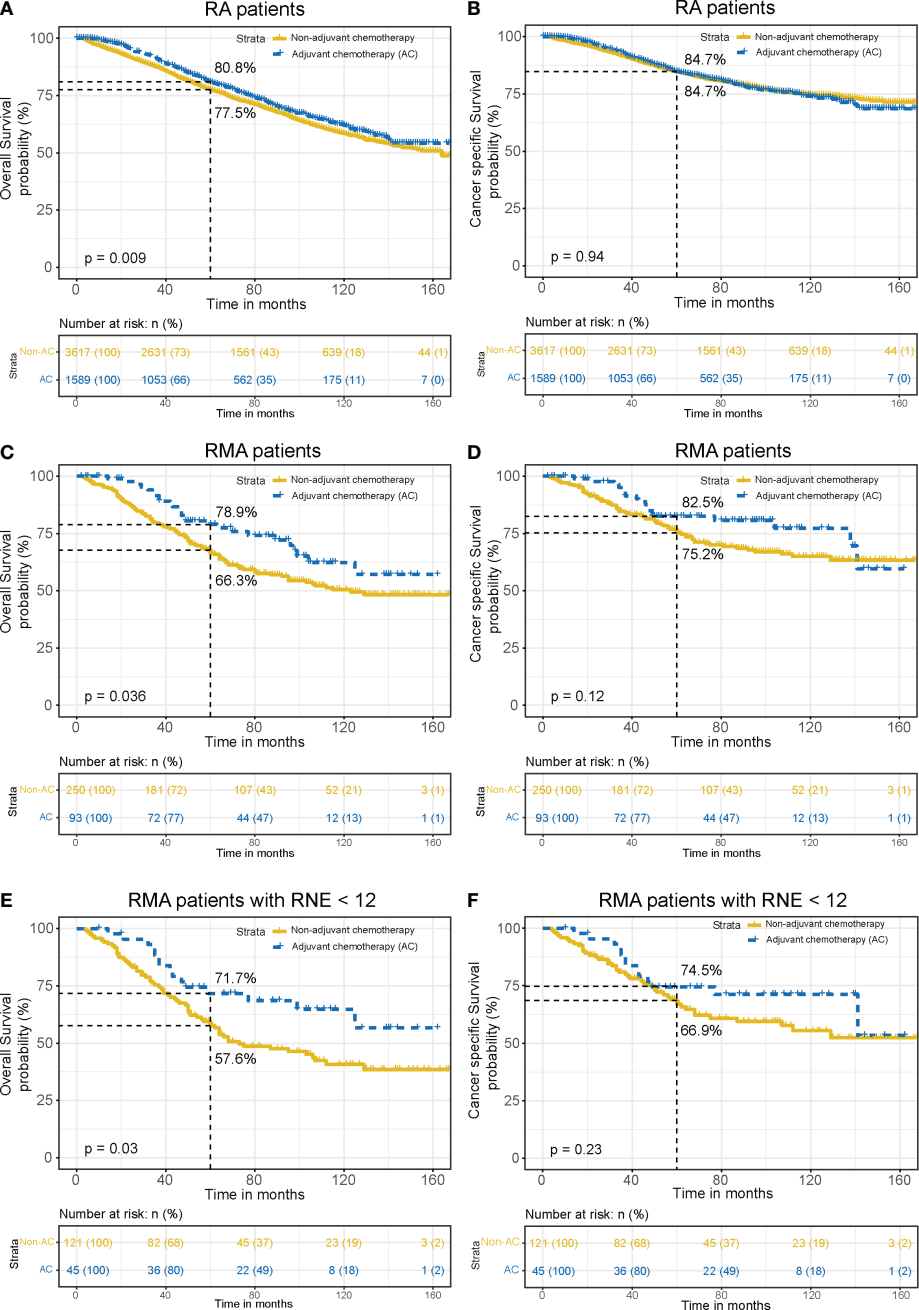
Pathological classification-stratified analysis. Comparison of OS **(A)** and CSS **(B)** between the AC group and non-AC group in RA patients. Comparison of OS **(C)** and CSS **(D)** between the AC group and non-AC group in RMA patients. Comparison of OS **(E)** and CSS **(F)** between the AC group and non-AC group in RMA patients with RNE < 12.

Further stratified analysis revealed that AC could improve the OS of RA patients (5-year OS: 80.8% with AC vs. 77.5% non-AC, *P* = 0.009), with a 3.3% absolute survival benefit at 5 years (**Figure 4A**). In RMA patients, AC could also improve the OS (5-year OS: 78.9% with AC vs. 66.3% non-AC, *P* = 0.036). The 5-year absolute survival benefit is as high as 12.6% (**Figure 4C**). Consistent results were observed in RNE < 12 subgroup (*P* = 0.03, **Figure 4E**). However, AC does not improve the CSS for RA patients (*P* = 0.94, **Figure 4B**), RMA patients (*P* = 0.12, **Figure 4D**) and RMA patients with RNE < 12 (*P* = 0.23, **Figure 4F**). The above results indicate that AC could significantly improve the OS of stage II RMA patients. However, the application of AC is likely to introduce toxicity implications for patients. Therefore, selectively administering AC to patients at high risk of distant recurrence could maximize the avoidance of unnecessary toxicity for patients.

Among all stage II RMA patients, those receiving AC were younger (*P* = 0.048) and had larger tumor sizes (*P* = 0.042) compared to those without AC (**Table 4**). Subsequently, to eliminate the impact of AC on RMA patients’ OS, RMA patients without AC were incorporated into the Cox proportional hazard model. In the univariate analysis, age, gender, pathologic T stage, RNE, and tumor size exhibited associations with OS (all *P* < 0.05, **Table 5**). Meanwhile, in the multivariate analysis, age, gender, pathologic T stage, RNE, and tumor size were all identified as independent prognostic factors (all *P* < 0.05, **Table 5**).

**Table 4:** Baseline characteristics of patients with pathologic stage II RMA.

Variables	Non-AC[n(%)] n=250	AC[n(%)] n=93	*P* value
Age			**0.048**
<65	137 (54.8%)	62 (66.7%)	
≥65	113 (45.2%)	31 (33.3%)	
Gender			0.951
Female	95 (38.0%)	35 (37.6%)	
Male	155 (62.0%)	58 (62.4%)	
Race			0.063
Non-white	40 (16.0%)	23 (24.7%)	
White	210 (84.0%)	70 (75.3%)	
Marital status			0.400
Single	40 (16.0%)	13 (14.0%)	
Married	201 (80.4%)	79 (84.9%)	
Unknown	9 (3.6%)	1 (1.1%)	
Household income			0.568
<$65,000	135 (54.0%)	47 (50.5%)	
≥$65,000	115 (46.0%)	46 (49.5%)	
Pathological T			0.156
T3	189 (75.6%)	77 (82.8%)	
T4	61 (24.4%)	16 (17.2%)	
RNE			0.998
<12	121 (48.4%)	45 (48.4%)	
≥12	129 (51.6%)	48 (51.6%)	
Tumor size			**0.042**
<5	126 (50.4%)	33 (35.5%)	
≥5	74 (29.6%)	38 (40.9%)	
Unknown	50 (20.0%)	22 (23.7%)	

*P* values in bold indicate *P* < 0.05.

**Table 5:** Univariate and multivariate Cox regression analysis for OS in RMA patients who did not receive AC.

Variables	Univariate analysis		Multivariate analysis	
	HR (95% CI)	*P* value	HR (95% CI)	*P* value
Age		**0.001**		**0.001**
<65	Reference		Reference	
≥65	1.935 (1.319-2.838)		1.679 (1.133-2.489)	
Gender		**0.027**		**0.020**
Female	Reference		Reference	
Male	1.590 (1.055-2.398)		1.637 (1.079-2.483)	
Race		0.830		
Non-white	Reference			
White	0.946 (0.570-1.570)			
Marital status				
Single	Reference			
Married	0.833 (0.507-1.368)	0.470		
Unknown	0.161 (0.022-1.204)	0.075		
Household income		0.710		
<$65,000	Reference			
≥$65,000	0.931 (0.637-1.359)			
Pathologic T		**0.006**		**0.031**
T3	Reference		Reference	
T4	1.795 (1.185-2.718)		1.603 (1.044-2.461)	
RNE		**0.003**		**0.014**
<12	Reference		Reference	
≥12	0.560 (0.381-0.824)		0.603 (0.403-0.902)	
Tumor size				
<5	Reference		Reference	
≥5	1.479 (1.087-2.220)	**0.037**	1.616 (1.068-2.447)	**0.023**
Unknown	0.504 (0.264-1.099)	0.059	0.535 (0.280-1.022)	0.058

*P* values in bold indicate *P* < 0.05.

### Constructing the nomogram and testing the effectiveness

**Figure 5:**
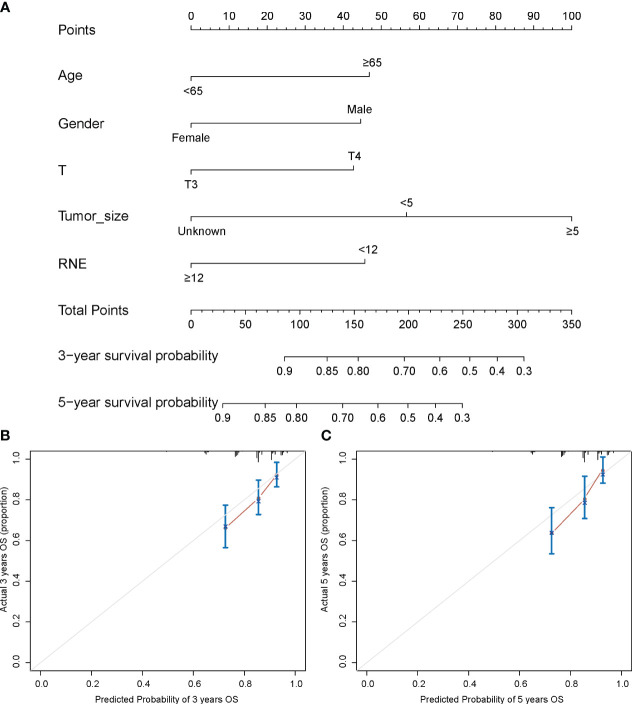
Nomogram for prediction of 3- and 5-year survival among stage II RMA patients **(A)**. Calibration curves for 3- **(B)** and 5- **(C)** year OS prediction.

A nomogram was constructed utilizing the independent prognostic factors identified through multivariate Cox regression analysis to predict 3- and 5-year OS rates, concurrently yielding a risk score for each RMA patient (**Figure 5A**). The C-index of the nomogram is 0.701 (95% CI: 0.652–0.750). The calibration curves of the nomogram for 3- and 5-year OS indicated that the predicted survival probabilities aligned closely with the actual survival probabilities (**Figures 5B, C**). The above results indicate that our nomogram possesses strong predictive potential and a high level of reliability.

### Overall cohort risk stratification system

**Figure 6:**
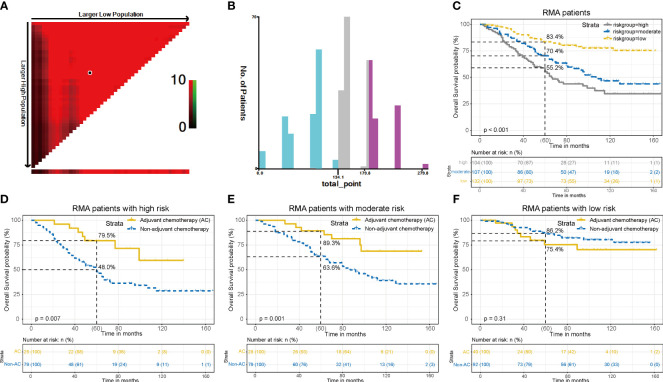
Overall RMA patients risk stratification system. **(A)**. The ideal threshold values. **(B)**. The number of patients in different risk subgroups. **(C)**. OS curves in low, moderate and high-risk groups of all patients. Compare OS between the AC and non-AC patients in high-risk group **(D)**, moderate-risk group **(E)**, and low-risk group **(F)**.

Using X-tile software, all RMA patients were divided into three subgroups based on their risk scores: low-risk (risk score: < 135, n = 132), moderate-risk (risk score: 136-182, n = 107), and high-risk (risk score: > 182, n = 104) (**Figures 6A, B**). The 5-year OS rates for the low, moderate, and high-risk groups were 83.4%, 70.4%, and 55.2%, respectively, with significant differences (*P* < 0.001) (**Figure 6C**). Subgroup analysis showed that in the high-risk group, AC significantly improved 5-year OS (79.5% with AC vs. 48.0% without AC, *P* = 0.007), yielding a 31.5% absolute survival benefit at 5 years (**Figure 6D**). Similar results were observed in the moderate-risk group, where AC led to a significant improvement in 5-year OS (89.3% with AC vs. 63.6% without AC, *P* = 0.009), with a 25.7% absolute survival benefit at 5 years (**Figure 6E**). However, in the low-risk group, no notable difference in OS was observed between patients who received AC and those who did not (86.2% with AC vs. 75.4% without AC, *P* = 0.31) (**Figure 6F**). The above findings suggest that stage II RMA patients in the high and moderate-risk groups could benefit from AC, while those in the low-risk group do not derive a benefit from AC.

### Validation of the risk stratification system

**Figure 7:**
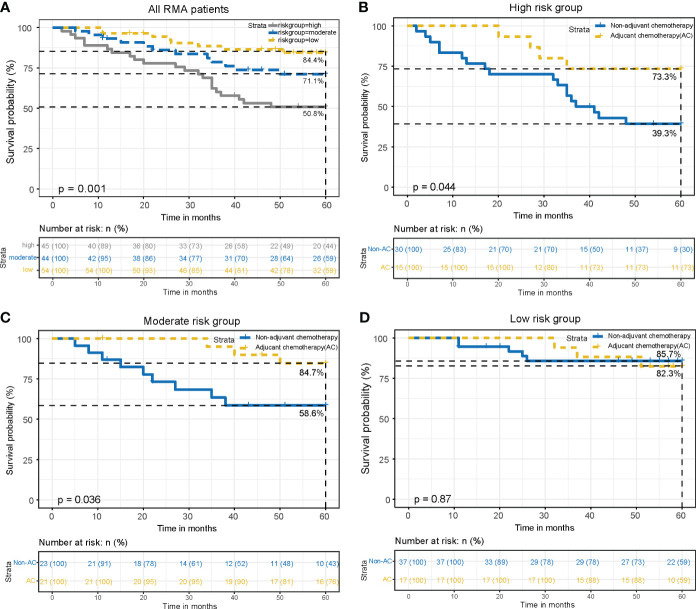
Validation of the risk stratification system. **(A)**. OS curves in low, moderate and high-risk groups of all RMA patients. Compare OS between the AC and non-AC patients in high-risk group **(B)**, moderate-risk group **(C)**, and low-risk group **(D)**.

Subsequently, the risk stratification system was verified for predictive accuracy in the external validation cohort. Significant differences in 5-year OS rates were observed among the low (84.4%), moderate (71.1%), and high-risk (50.8%) groups (*P* = 0.001) (**Figure 7A**). In the high-risk subgroup, AC significantly improved 5-year OS (73.3% with AC vs. 39.3% without AC, *P* = 0.044), with an absolute survival benefit of 34.0% at 5 years (**Figure 7B**). Similarly, in the moderate-risk subgroup, AC led to a significant improvement in 5-year OS (84.7% with AC vs. 58.6% without AC, *P* = 0.036), with an absolute survival benefit of 26.1% at 5 years (**Figure 7C**). However, in the low-risk subgroup, no notable difference in OS was observed between patients who received AC and those who did not (82.3% with AC vs. 85.7% without AC, *P* = 0.87) (**Figure 7D**). The above results indicated that our risk stratification system was accurate.

## Discussion

Although numerous guidelines currently recommend NCR followed by TME surgery for locally advanced rectal cancer patients (8, 13), the question of whether stage II RMA patients require additional AC after surgery remains a subject of debate. Some guidelines recommend routine AC for stage II rectal cancer patients after surgery (8), but the benefits of AC for stage II rectal cancer are primarily extrapolated from clinical trials conducted in colon cancer (14–16). The exact benefits of AC following NCR for rectal cancer remain unclear. Sainato A, and Breugom AJ, et al. (17, 18) found that NCR followed by AC after TME did not show significant benefits in OS, disease-free survival (DFS), and recurrence rate in patients with locally advanced rectal cancer. Glynne-Jones R, et al. ‘s study (19) found that in the AC group (n=54), there was no statistically significant improvement observed in the 3-year DFS (78% vs. 71%, p=0.56) and OS (89% vs. 88%, p=0.75) compared to the non-AC group (n=59). However, this study had a limited sample size and was prematurely terminated, so further clinical trials are needed to confirm the role of AC. Hence, it remains uncertain whether AC is warranted for all stage II rectal cancer patients who have received NCR and undergone TME.

RMA is a distinct pathological subtype of rectal cancer. While its treatment strategies resemble those of rectal cancer, its prognosis tends to be worse due to its lower sensitivity to chemotherapy (20). However, clinical research from various centers debates whether AC benefits RMA patients. In our study, we found that RA has longer OS and CSS than RMA in the all and non-AC stage II patients, but no significant difference in OS and CSS were observed between the RA and RMA groups for receiving AC treatment. PSM analyses yielded similar results. Stratified analysis found that AC could both improve the OS of RA patients and RMA patients (the absolute survival benefits were 3.3% and 12.6% at 5 years) patients. Therefore, AC is an essential measure for stage II RMA patients after NCR and TME (21). However, the application of postoperative AC has both a cytotoxic effect on residual tumor lesions and potential toxicity to patients (22, 23). Therefore, employing risk stratification to selectively treat patients at a high risk of distant recurrence can enhance clinical decision-making and prevent unnecessary toxicity in those unlikely to benefit.

In recent years, there have been rapid advancements in survival prediction models and risk stratification tools (24). Currently, numerous prediction models and risk stratification systems are being used for prognostic assessment or clinical treatment guidance in various cancers, including gastric cancer (25, 26), bladder cancer (27, 28), lung cancer (29, 30), nasopharyngeal carcinoma (31, 32), and more. Although prediction models cannot serve as a substitute for the evidence obtained from clinical trials, they hold significant value in supplying information for guiding clinical decisions in cancers with limited clinical trial data. Therefore, we established a nomogram based on independent prognostic factors identified through multivariate Cox regression analysis to predict 3- and 5-year OS rates, concurrently yielding a risk score for each patient. Using X-tile software, all RMA patients were divided into three subgroups based on their risk scores. KM curves and log-rank tests indicate that the high-risk group has the poorest prognosis, while the low-risk group has the best prognosis. This demonstrates the effectiveness of our risk stratification system. Further subgroup analysis results indicate that in the moderate and high-risk groups, receiving AC treatment significantly improves the 5-year OS of patients. However, in the low-risk group, there is no significant difference in OS between patients who received AC and those who did not. This result suggests that patients in the high and moderate-risk groups can benefit from AC, while those in the low-risk group do not derive a benefit from AC. And the external validation cohort yielded similar results, indicating the accuracy of our risk stratification system. Therefore, recommend AC for moderate and high-risk patients, while low-risk patients may forego it.

Finally, this study also has some limitations. First, the number of patients with stage II RMA who received NCR, underwent TME, and subsequently received AC is limited (n=93), which could potentially impact the assessment of the effectiveness of AC. Secondly, the specific regimens and completion rates of NCR and AC in patients were not clearly defined, which could introduce bias into our results. Thirdly, this study is retrospective, which may introduce bias as well. However, our study provides guidance on whether additional AC is necessary for stage II RMA following NCR and TME. Our model holds significant implications for personalized treatment of stage II RMA patients, highlighting the importance of our findings.

## Conclusion

In summary, our population-based retrospective cohort study suggests that, compared to RA, stage II RMA benefits more from AC after NCR. Meanwhile, based on our prognostic predicted model and risk stratification system, it is advisable for moderate and high-risk RMA patients with pathological stage II after NCR undergo AC, while low-risk patients do not need AC.

## Data availability statement

Publicly available datasets were analyzed in this study. This data can be found here: https://seer.cancer.gov/.

## Ethics statement

The studies involving humans were approved by the ethics committee of First Affiliated Hospital of Nanchang University. The studies were conducted in accordance with the local legislation and institutional requirements. The participants provided their written informed consent to participate in this study.

## Author contributions

YHL: Data curation, Funding acquisition, Methodology, Software, Validation, Writing – original draft. HL: Data curation, Methodology, Writing – original draft, Investigation. HS: Investigation, Methodology, Writing – original draft. TL: Investigation, Writing – original draft, Validation. YXL: Writing – original draft, Data curation, Software. YLY: Writing – original draft, Investigation, Methodology. ML: Methodology, Writing – original draft, Data curation. AL: Data curation, Writing – original draft, Investigation, Software. YL: Data curation, Writing – original draft, Methodology, Validation. YY: Data curation, Methodology, Validation, Writing – original draft. TYL: Conceptualization, Supervision, Writing – review & editing.
