# Supplementary material for: Risk stratification of stage II rectal mucinous adenocarcinoma to predict the benefit of adjuvant chemotherapy following neoadjuvant chemoradiation and surgery
Source: Front Oncol. 2024 Mar 5;14:1352660. doi: 10.3389/fonc.2024.1352660 (PMC10952835; doi:10.3389/fonc.2024.1352660)
Supplement: Supplementary file 1 [file Table_1.docx]

Supplementary table 1. Univariate and multivariate Cox regression analyses of CSS for the stage II patients with RA and RMA

| Variables | Univariate analysis | |  | Multivariate analysis | |
| --- | --- | --- | --- | --- | --- |
|  | HR (95% CI) | *P* value |  | HR (95% CI) | *P* value |
| Age |  | **< 0.001** |  |  | **< 0.001** |
| <65 | Reference |  |  | Reference |  |
| ≥65 | 1.423 (1.256-1.614) |  |  | 1.441 (1.268-1.636) |  |
| Gender |  | 0.433 |  |  |  |
| Female | Reference |  |  |  |  |
| Male | 1.053 (0.925-1.198) |  |  |  |  |
| Race |  | 0.921 |  |  |  |
| Non-white | Reference |  |  |  |  |
| White | 0.992 (0.843-1.167) |  |  |  |  |
| Marital status |  |  |  |  |  |
| Single | Reference |  |  | Reference |  |
| Married | 0.830 (0.703-0.979) | **0.027** |  | 0.765 (0.647-0.904) | **0.002** |
| Unknown | 0.943 (0.649-1.368) | 0.755 |  | 0.843 (0.580-1.225) | 0.370 |
| Household income |  | **0.006** |  |  | **0.004** |
| <$65,000 | Reference |  |  | Reference |  |
| ≥$65,000 | 0.839 (0.740-0.951) |  |  | 0.833 (0.735-0.944) |  |
| Pathologic T |  | 0.068 |  |  |  |
| T3 | Reference |  |  |  |  |
| T4 | 1.180 (0.988-1.408) |  |  |  |  |
| Adjuvant chemotherapy |  | 0.579 |  |  |  |
| Non-AT | Reference |  |  |  |  |
| AT | 0.961 (0.835-1.106) |  |  |  |  |
| RNE |  | **< 0.001** |  |  | **< 0.001** |
| <12 | Reference |  |  | Reference |  |
| ≥12 | 0.691 (0.610-0.783) |  |  | 0.707 (0.624-0.801) |  |
| Pathological classification |  | **0.002** |  |  | **0.004** |
| RA | Reference |  |  | Reference |  |
| RMA | 1.420 (1.142-1.765) |  |  | 1.373 (1.104-1.707) |  |
| Tumor size |  |  |  |  |  |
| <5 | Reference |  |  |  |  |
| ≥5 | 1.077 (0.930-1.246) | 0.322 |  |  |  |
| Unknown | 1.096 (0.936-1.285) | 0.256 |  |  |  |
