# Supplementary material for: Risk stratification of stage II rectal mucinous adenocarcinoma to predict the benefit of adjuvant chemotherapy following neoadjuvant chemoradiation and surgery
Source: Front Oncol. 2024 Mar 5;14:1352660. doi: 10.3389/fonc.2024.1352660 (PMC10952835; doi:10.3389/fonc.2024.1352660)
Supplement: Supplementary file 2 [file Table_2.docx]

| Variables | RA[n(%)]  n=343 | RMA[n(%)]  n=343 | *P* value |
| --- | --- | --- | --- |
| Age |  |  | 0.757 |
| <65 | 195(56.9%) | 199(58.0%) |  |
| ≥65 | 148 (43.1%) | 144(42.0%) |  |
| Gender |  |  | 0.875 |
| Female | 128(37.3%) | 130(37.9%) |  |
| Male | 215(62.7%) | 213(62.1%) |  |
| Race |  |  | 0.922 |
| Non-white | 64(18.7%) | 63(18.4%) |  |
| White | 279(81.3%) | 280(81.6%) |  |
| Marital status |  |  | 0.498 |
| Single | 45(13.1%) | 53(15.5%) |  |
| Married | 291(84.8%) | 280(81.6%) |  |
| Unknown | 7(2.0%) | 10(2.9%) |  |
| Household income |  |  | 0.878 |
| <$65,000 | 184(53.6%) | 182(53.1%) |  |
| ≥$65,000 | 159(46.4%) | 161(46.9%) |  |
| Pathological T |  |  | 0.702 |
| T3 | 270(78.7%) | 266(77.6%) |  |
| T4 | 73(21.3%) | 77(22.4%) |  |
| Adjuvant chemotherapy |  |  | 0.932 |
| Non-AT | 249(72.6%) | 250(72.9%) |  |
| AT | 94(27.4%) | 93(27.1%) |  |
| RNE |  |  | 0.939 |
| <12 | 167(48.7%) | 166(48.4%) |  |
| ≥12 | 176(51.3%) | 177(51.6%) |  |
| Tumor size |  |  | 0.972 |
| <5 | 162(47.2%) | 159(46.3%) |  |
| ≥5 | 111(32.4%) | 112(32.7%) |  |
| Unknown | 70(20.4%) | 72(21.0%) |  |

Supplementary table 2. Baseline characteristics of pathologic stage II patients with RA and RMA after PSM.
