# Supplementary material for: Risk stratification of stage II rectal mucinous adenocarcinoma to predict the benefit of adjuvant chemotherapy following neoadjuvant chemoradiation and surgery
Source: Front Oncol. 2024 Mar 5;14:1352660. doi: 10.3389/fonc.2024.1352660 (PMC10952835; doi:10.3389/fonc.2024.1352660)
Supplement: Supplementary file 3 [file Table_3.docx]

Supplementary table 3. Univariate and multivariate Cox regression analyses of CSS for the stage II patients with RA and RMA after PSM

| Variables | Univariate analysis | |  | Multivariate analysis | |
| --- | --- | --- | --- | --- | --- |
|  | HR (95% CI) | *P* value |  | HR (95% CI) | *P* value |
| Age |  | 0.080 |  |  |  |
| <65 | Reference |  |  |  |  |
| ≥65 | 1.326 (0.967-1.816) |  |  |  |  |
| Gender |  | 0.682 |  |  |  |
| Female | Reference |  |  |  |  |
| Male | 0.935 (0.677-1.291) |  |  |  |  |
| Race |  | 0.130 |  |  |  |
| Non-white | Reference |  |  |  |  |
| White | 1.426 (0.900-2.260) |  |  |  |  |
| Marital status |  |  |  |  |  |
| Single | Reference |  |  |  |  |
| Married | 0.846 (0.547-1.309) | 0.452 |  |  |  |
| Unknown | 0.382 (0.090-1.618) | 0.192 |  |  |  |
| Household income |  | 0.093 |  |  |  |
| <$65,000 | Reference |  |  |  |  |
| ≥$65,000 | 0.760 (0.552-1.046) |  |  |  |  |
| Pathologic T |  | 0.142 |  |  |  |
| T3 | Reference |  |  |  |  |
| T4 | 1.326 (0.910-1.932) |  |  |  |  |
| Adjuvant chemotherapy |  | 0.501 |  |  |  |
| Non-AT | Reference |  |  |  |  |
| AT | 0.882 (0.612-1.272) |  |  |  |  |
| RNE |  | **< 0.001** |  |  | **< 0.001** |
| <12 | Reference |  |  | Reference |  |
| ≥12 | 0.507 (0.365-0.703) |  |  | 0.507 (0.365-0.703) |  |
| Pathological classification |  | **0.003** |  |  | **0.003** |
| RA | Reference |  |  | Reference |  |
| RMA | 1.612 (1.172-2.219) |  |  | 1.612 (1.171-2.218) |  |
| Tumor size |  |  |  |  |  |
| <5 | Reference |  |  |  |  |
| ≥5 | 0.945 (0.651-1.371) | 0.764 |  |  |  |
| Unknown | 1.257 (0.845-1.871) | 0.260 |  |  |  |
